# Reply: “Comment on Search and Selection of Probiotics that Improve Mucositis Symptoms in Oncologic Patients. A Systematic Review. Nutrients 2019, *11*, 2322”

**DOI:** 10.3390/nu12020401

**Published:** 2020-02-03

**Authors:** José Antonio Picó-Monllor, José Manuel Mingot-Ascencao

**Affiliations:** 1 Deparment of Pharmacology, Pediatrics and Organic Chemistry, Faculty of Pharmacy, Universidad Miguel Hernández, 03200 Elche, Spain; 2 Korott, s.l., 03801 Alcoy, Spain; jmmingot@korott.com

In the article, “Search and Selection of Probiotics That Improve Mucositis Symptoms in Oncologic Patients. A Systematic Review” [1] which appears in Volume 11, Issue 10 of Nutrients, there is a correction on the official term of the drug name used in the article.

As De Simone [2] points out, The drug term VSL#3^®^ is replaced with the generic name of the formulation, “De Simone formulation”, following a legal dispute between the distributors of the product VSL#3^®^ and the formula inventor.

This change does not affect the study of the paper.
